# Pre-Solutrean rock art in southernmost Europe: Evidence from Las Ventanas Cave (Andalusia, Spain)

**DOI:** 10.1371/journal.pone.0204651

**Published:** 2018-10-17

**Authors:** Miguel Cortés-Sánchez, José Antonio Riquelme-Cantal, María Dolores Simón-Vallejo, Rubén Parrilla Giráldez, Carlos P. Odriozola, Lydia Calle Román, José S. Carrión, Guadalupe Monge Gómez, Joaquín Rodríguez Vidal, Juan José Moyano Campos, Fernando Rico Delgado, Juan Enrique Nieto Julián, Daniel Antón García, M. Aránzazu Martínez-Aguirre, Fernando Jiménez Barredo, Francisco N. Cantero-Chinchilla

**Affiliations:** 1 Departamento de Prehistoria y Arqueología, University of Sevilla, Sevilla, Spain; 2 HUM-949, Tellus, Prehistoria y Arqueología en el sur de Iberia, University of Sevilla, Sevilla, Spain; 3 ICArEHB-Interdisciplinary Center for Archaeology and Evolution of Human Behaviour - FCHS da Universidade do Algarve, Faro, Portugal; 4 Departamento de Geografía y Ciencias del Territorio, University of Córdoba, Córdoba, Spain; 5 Servicio de Microanálisis, Centro de Investigación y Tecnología de la Universidad de Sevilla (CITIUS), University of Sevilla, Sevilla, Spain; 6 Departamento de Biología Vegetal, University of Murcia, Murcia, Spain; 7 RNM349 Group, Mineralogía y Geoquímica Ambiental y de la Salud, Departamento de Cristalografía, Mineralogía y Química Agrícola, University of Sevilla, Sevilla, Spain; 8 Departamento de Ciencias de la Tierra, University of Huelva, Huelva, Spain; 9 Departamento de Expresión Gráfica e Ingeniería en la Edificación, University of Sevilla, Sevilla, Spain; 10 Departamento de Física Aplicada, Escuela Técnica Superior de Ingeniería Agronómica, University of Sevilla, Sevilla, Spain; 11 Centro Nacional de Investigación sobre la Evolución Humana, CENIEH, Burgos, Spain; 12 Water Engineering and Environmental group, University of Southampton, Southampton, United Kingdom; University at Buffalo - The State University of New York, UNITED STATES

## Abstract

The south of Iberia conserves an important group of Palaeolithic rock art sites. The graphisms have been mostly attributed to the Solutrean and Magdalenian periods, while the possibility that older remains exist has provoked extensive debate. This circumstance has been linked to both the cited periods, until recently, due to the transition from the Middle to Upper Palaeolithic in the extreme southwest of Europe as well as the non-existence of some of the early periods of Palaeolithic art documented in northern Iberia. This study presents the results of interdisciplinary research conducted in Las Ventanas Cave. These results enabled us to identify a new Palaeolithic rock art site. The technical, stylistic and temporal traits point to certain similarities with the range of exterior deep engravings in Cantabrian Palaeolithic rock art. Ventanas appears to corroborate the age attributed to those kinds of graphic expression and points to the early arrival of the Upper Palaeolithic in the south of Iberia. Importantly, the results provide information on the pre-Solutrean date attributed to trilinear hind figures. These findings challenge the supposed Neanderthal survival idea at one of the main late Middle Palaeolithic southern Iberian sites (Carigüela) and, due to the parallels between them and an engraving attributed to this period in Gibraltar, it raises the possibility of interaction between modern humans and Neanderthals in the extreme southwest of Europe.

## Introduction

The south of the Iberian Peninsula is, together with the Cantabrian Region (northern coast of the Iberian Peninsula) and southern France, one of the areas with the largest number of caves with Palaeolithic rock art in the southwest of Europe. However, in these latter two, the graphic sequence seems to have begun earlier, spanning the entire development of the Upper Palaeolithic (UP) [1–5]. In this context, a series of caves with exterior graphics, also known as "first and second graphic horizons of the Nalón" [1, 6, 7] are among the oldest graphic expressions of Pleistocene art in western Europe, although their time span is also subject to some debate [8–10].

In the south of Iberia, historiography of the chronocultural Upper Pleistocene sequence generally, until recently, involved a lengthy Middle Palaeolithic (MP) and the very late arrival of the technocomplexes of the Early Upper Palaeolithic (EUP) to this region [11]. The impact of this model on the Palaeolithic art in this geographic area ensured that all the graphic images were attributed generally to the Solutrean and Magdalenian periods [12]. Only in recent years, the appearance of EUP levels at various sites [11, 13–16] and the reassessment of some art manifestations in various caves (e.g. Ardales, La Pileta, etc.) have enabled us to reconsider the existence of horizons attributable to this period in southernmost Iberia [17–22].

In this historiographical context, the new finds presented in this study constitute evidence for ancient graphic periods in southern Iberia, linkable chronoculturally to the EUP.

## The site

**Fig 1:**
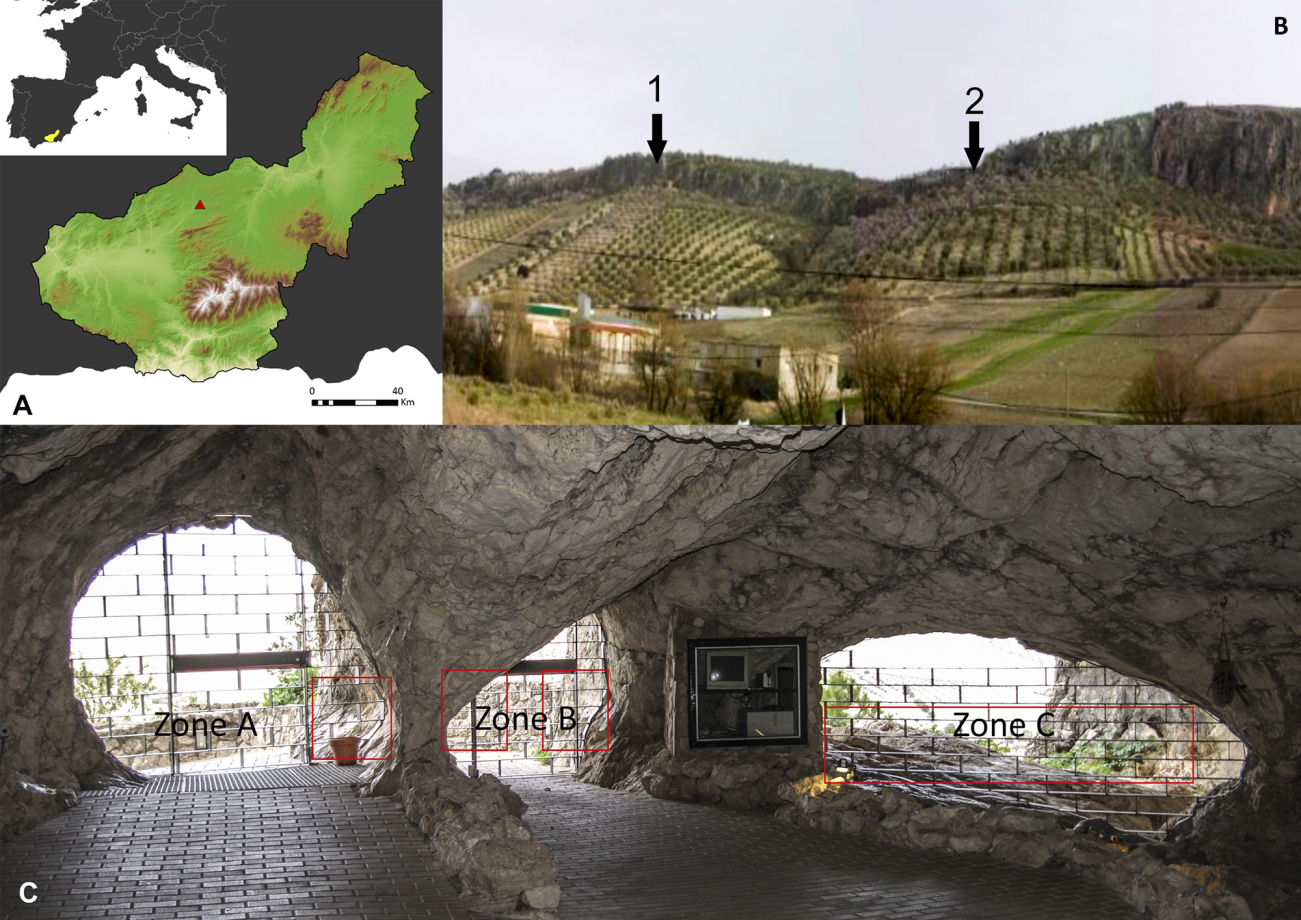
Geographical context and study areas. (A) Location within Iberia. (B) Location of Ventanas (1) and Carigüela (2). (C) View of the three entrances from inside with an indication of the engraved zones. Geographic data from Centro Nacional de Información Geográfica (Ministerio de Fomento, Spanish government) http://www.ign.es/web/ign/portal.

Ventanas is a cave located in the south of the Iberian Peninsula (Fig 1A) (lat. 37° 26′ 30″N, long. 3° 25′ 42″W), near the town of Píñar in the province of Granada (Spain). The site is situated at an altitude of 1,015 masl in the foothills of Sierra Harana (Fig 1B). The cave is 1,200m long with a vertical range of 37.5m between its highest and lowest points, and takes its name from its three entrances (Fig 1C).

From a geological point of view, the cave is in a Jurassic limestone formation, greatly affected by karst processes, belonging to the Baetic Ranges [23]. The cave entrance is located in a 20m high escarpment, due to a tectonic contact.

The most significant exokarst landform is a karren of rounded crests, while endokarst landforms include a wide variety of speleothems (columns, flowstones, draperies, stalagmites, stalactites and gours) and three different levels of galleries reflecting the speleogenetic history of the cave. The upper galleries correspond to the oldest times in which the endokarst was generated, and the lowest ones correspond to the most recent [24]. The intermediate level is formed by three small passages which coalesce into the great entrance gallery (Fig 1C).

The first references to the archaeology of Ventanas are attributable to H. Obermaier, who visited the cave and identified Neolithic remains. Later, J.-Ch. Sphani test-pitted it in 1954 in the search for Palaeolithic remains and, failing to find results, moved on to the nearby cave of Carigüela (Fig 1B call 1). After years of neglect and decline, in late 1996 the cave was gated and Ventanas was opened to tourists. In the course of this work, several artefacts were recovered which define a technocultural sequence of the Upper Palaeolithic (UP), Recent Prehistory, the Middle Ages and the Contemporary Era [25].

## Methodology

**Fig 2:**
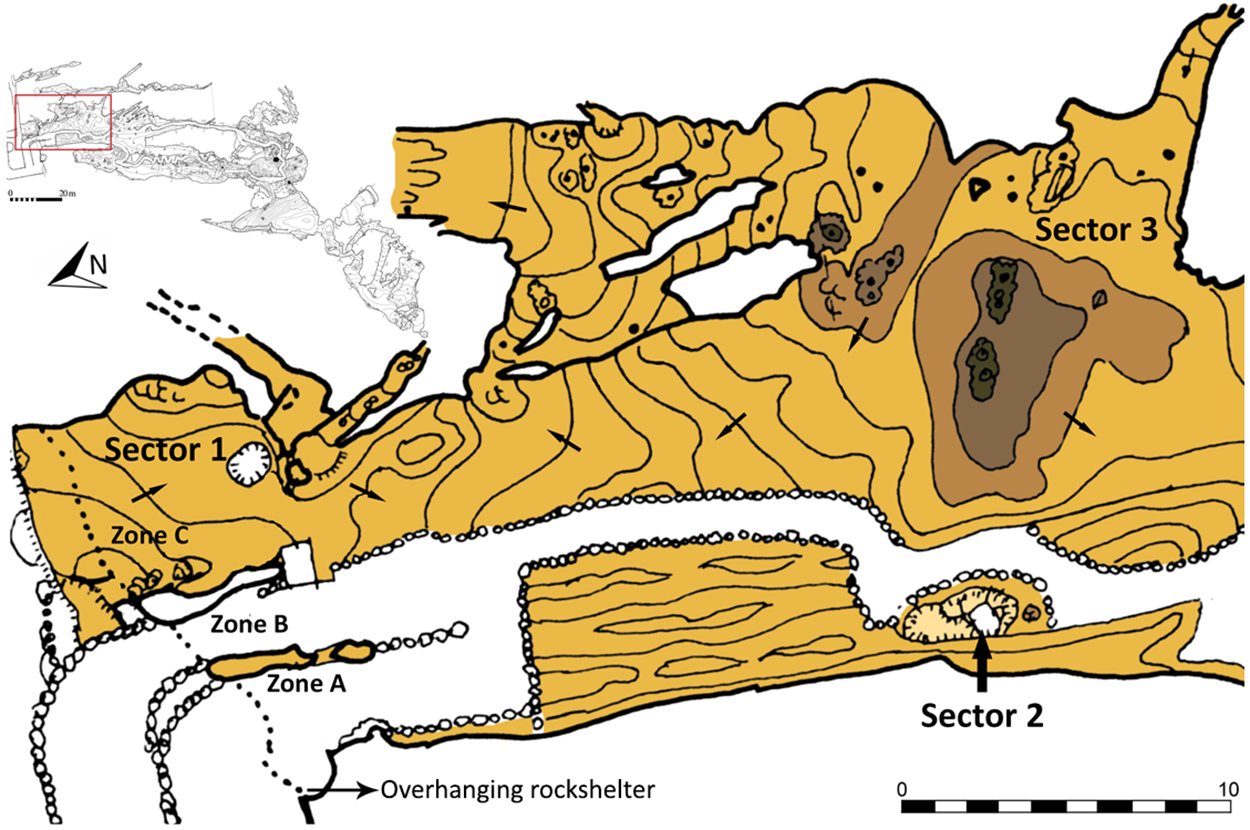
Topography of Ventanas (small) with zoomed-in view of the area with the engravings and indication of the location of zones A, B and C.

**Fig 3:**
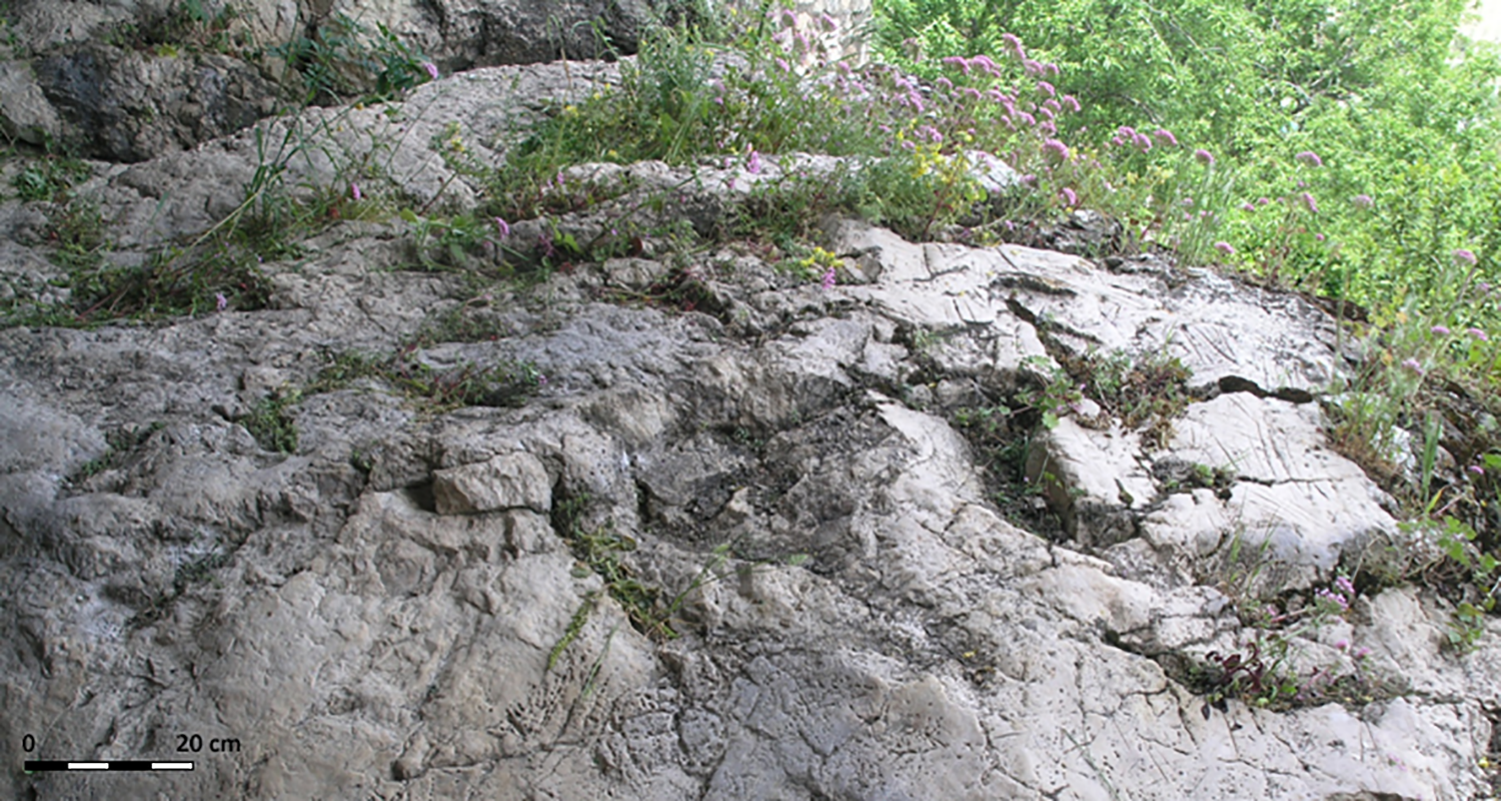
State of the engravings panel at the start of the research (zone C).

The fieldwork focused on the outermost areas of the intermediate level (Fig 2) whilst a more general investigation was conducted inside the cave. During this fieldwork, rock art was only discovered at the entrances that receive direct sunlight (zones A, B and C, (Figs 1C and 3) areas which are discussed below. The rock art basically consists of engravings. These were found covered by herbaceous vegetation which was growing in sediment that filled cracks and fissures in the limestone surface (Fig 3). These plants prevented the adequate documentation of the graphic art, and were removed with scissors to avoid pulling out the roots and the sediment attached to them. Later, using non-metal tools, the sediment that covered the gaps was removed in order to demarcate and adequately document the engravings. This sediment was sifted using a mesh size of 0.1mm and preserved in PVC bags. Furthermore, in some areas, the engravings are superficially covered with lichen and carbon, which form a thin biofilm that covers the engravings but does not prevent a full reading being conducted, so they were not removed.

### 2D and 3D documentation

Zones A, B and C (Figs 1C and 2) were recorded in a georeferenced 3D model by means of a Leica ScanStation C10 laser scanner in three correlated positions. The data collected from specific sectors–those containing engravings–were obtained precisely with a handheld Artec MHT 3D scanner. The scanning speed remained invariable during the process, overlapping adjacent readings according to the light pulse matrix of the surface. The focus distance was limited to the 500-700mm interval and controlled in real time through the histogram using Artec Studio 10 Professional software.

**Fig 4:**
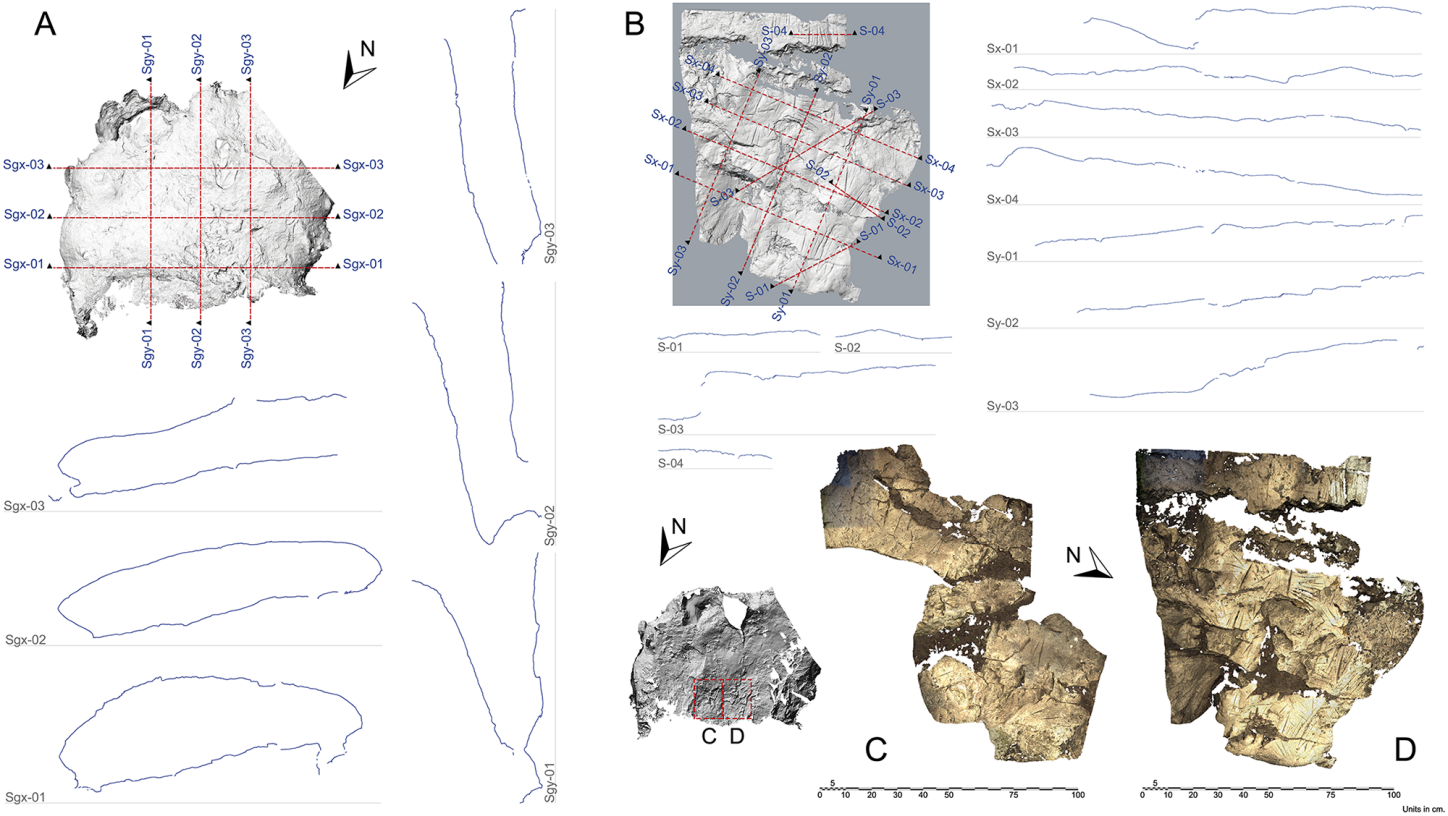
Zone C laser scan results. (A) Ortographic views. (B) Sections and geometry. (C and D) Textured 3D mesh.

The post-processing of the data from both techniques cited above took place subsequently. With regards to the handheld scanner, specific algorithms were applied to the models of each sector in order to optimise the resulting 3D meshes (Fig 4). For the laser scanning process, the data records were assembled using Leica Cyclone software. The global 3D model of the studied area was formed by overlapping common points in the data of the consecutive readings.

These sets of data points were filtered using the Rapidform software to reduce noise and to eliminate the elements preventing accurate readings of the geometry being accomplished by the scanner (Fig 4C and 4B). A triangulate mesh was next generated in order to make this geometry easier to manage. Finally, the 3D model with the top view, elevations and sections–with measurements–of the specific location in the cave was built using BIM (Building Information Modelling) software.

The engravings were documented using digital image processing and computer vision techniques with different software. Digital images were obtained remotely in RAW format using the Digicam Control (http://www.digicamcontrol.com/) software and Canon EOS 50F camera. Post-processing software used includes Darktable (https://www.darktable.org/), Image Composite Editor (https://www.microsoft.com/en-us/research/project/image-composite-editor/), Visual SFM (http://ccwu.me/vsfm/), CloudCompare (http://www.cloudcompare.org/), Blender (https://www.blender.org/), Hypercube (https://www.agc.army.mil/), ImageJ (https://imagej.nih.gov/ij) and GIMP (https://www.gimp.org/).

The 2D computing workflow consisted of classifying images by area, digital development, obtaining high-resolution photomosaics [26, 27] analysing Principal Components [28], local matrix operations, segmenting interesting characteristics and vectorisation of engravings. Several areas of interest were also analysed by generating 3D models [29] and mesh analysis.

### Characterization of pigments

In-situ elemental and molecular analyses of pigments in Ventanas were carried out directly, without applying any pre-treatment of the sample, using a mobile Raman spectrometer.

**Fig 5:**
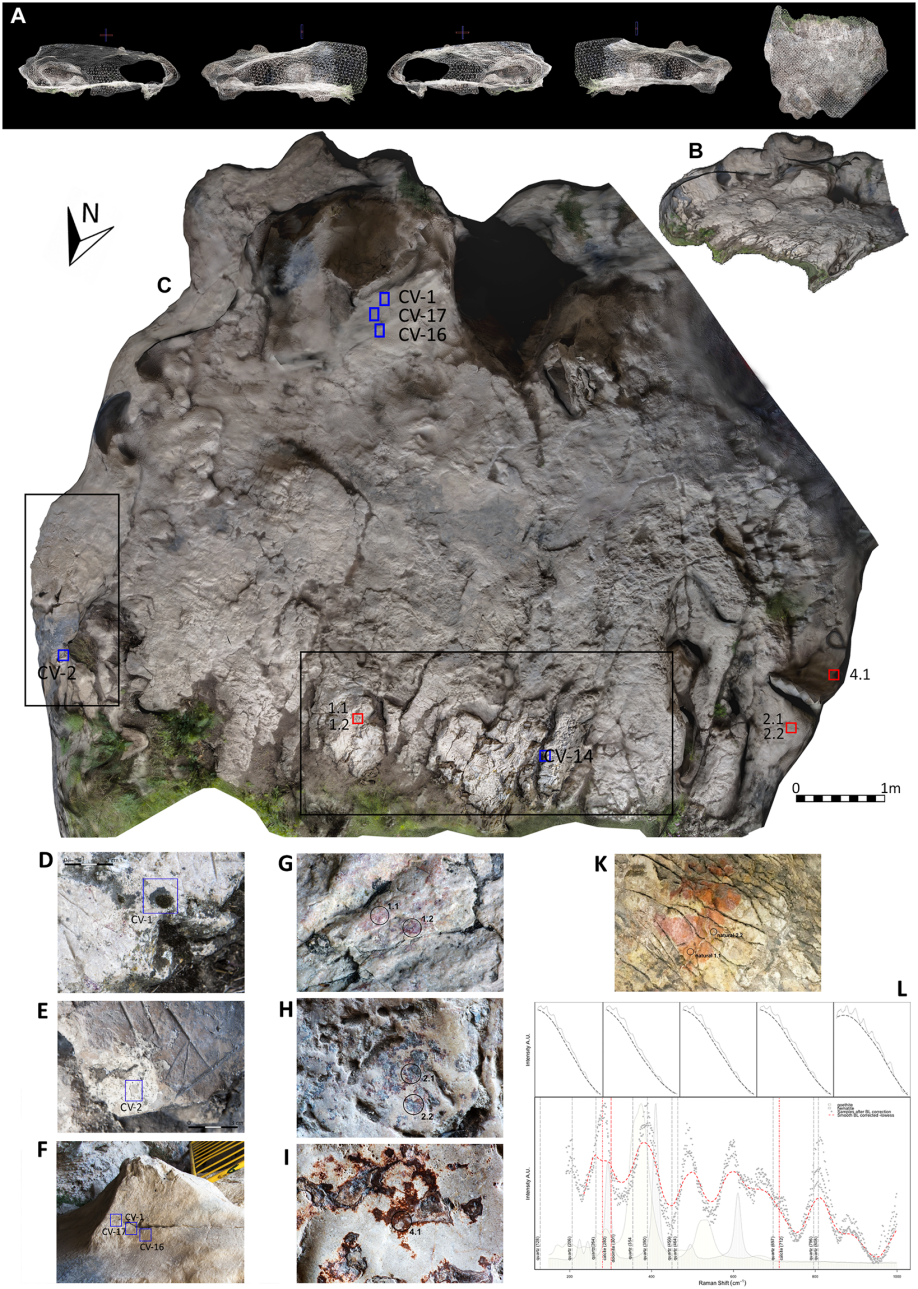
Zone C textured 3D model and location of analysed samples of pigments and dates. Black squares indicate main engravings areas, red squares pigment analysis samples and blue squares dating samples. (A) 3D mesh orthographic views. (B) Perspective view. (C) Top view of the floor. (D) Detail of CV-14 dating sample. (E) Detail of CV-2 dating sample. (F) Detail of CV-1, CV-16 and CV-17 dating samples. (G-H) Detail of analysed pigment samples. (K) Natural ochre from the cave. (L) Raman analysis plot.

Red-coloured areas in zones A, B and C (Fig 5G–5I) were analysed by Raman spectroscopy. This technique enables the identification of any archaeological materials (pigments, substrata, etc) since: (a) mineral polymorphs and compounds with different numbers of hydration molecules provide different Raman features in terms of position and relative intensity, and (b) amorphous compounds yield very good Raman spectra, and/or (c) both organic and inorganic materials present a Raman effect [30].

Raman analyses were performed using a portable BWTEK iRaman Plus spectrometer (B&WTEK, Inc., Newmark, USA) provided with a 785nm excitation laser. Since the laser output power can be adjusted with the software between 0–100%, the maximum power of the laser (420mW) was reduced (10% of the maximum) to avoid the photo-decomposition of the samples (burning) or phase transitions. No filters were used during measurements.

### Mineralogical characterization of weathering patinas

Weathering patinas were observed in Zone C (Figs 3 and 5D–5F) affecting both the parent rock and the engravings. Five samples were taken using disposable sterile scalpels (CV-1 to CV-5).

The mineral phases were identified using a D8 Advance A25 Bruker diffractometer. The software used was XPowder (See 2010.01.13 PRO), in conjunction with the ICDD database (International Centre for Diffraction Data). The semi-quantitative estimation of the mineral content was carried out using the intensity factors calculated [31, 32].

### Dating

**Fig 6:**
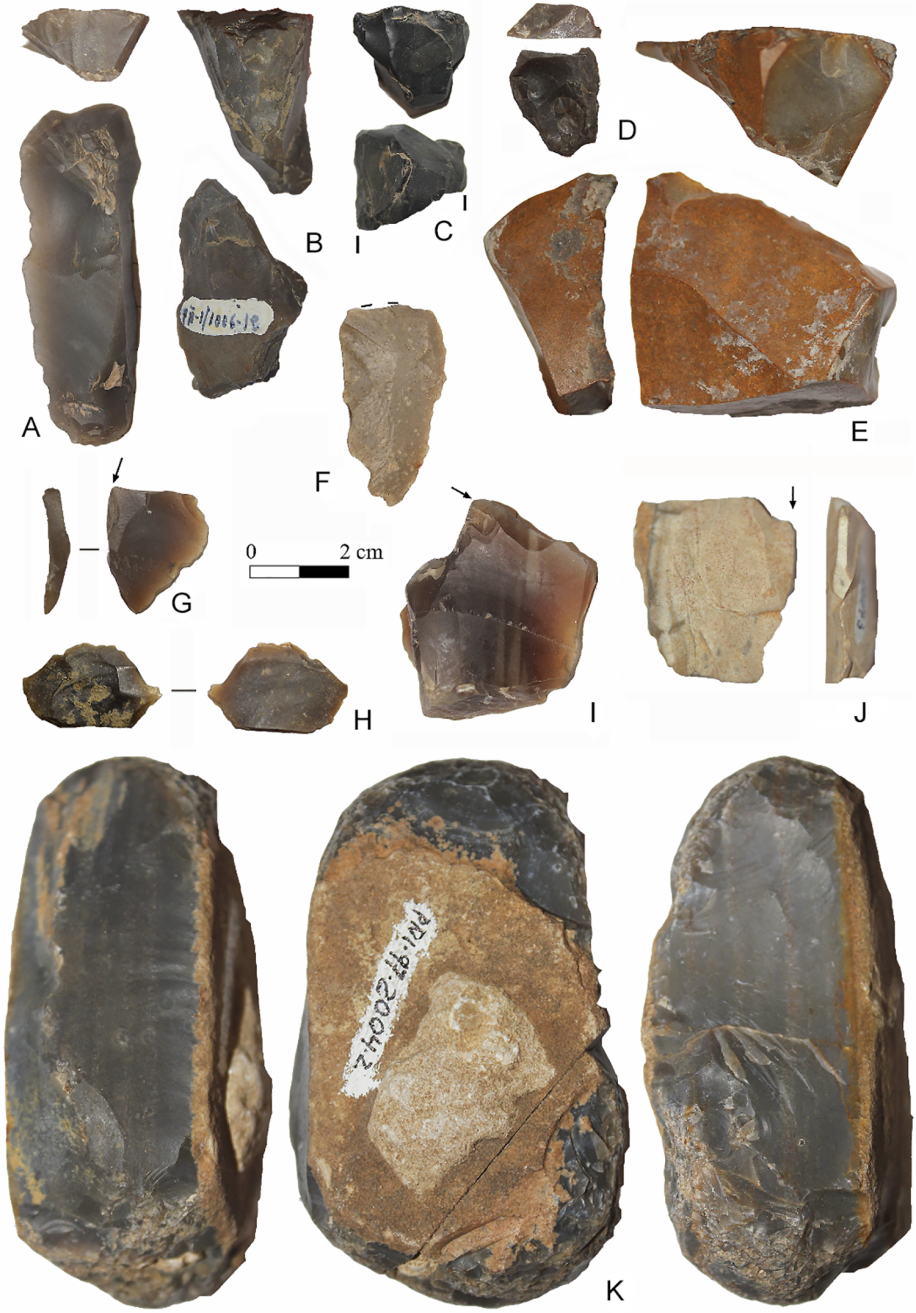
Lithic industries from Sector 1. (A-F) Endscrapers. (G-J) Burins. (K) Hammer on recycled blade core.

Four ^14^C/AMS dates in Table 1 were obtained to determine the chronology of the engravings. The first two dates correspond to the samples of patinas CV-1 and CV-2 which partially covered some engravings and the limestone edges affecting them (Fig 5D–5F) the third is the result of analysing the carbonates adhered to an endscraper from Zone C (Fig 6E). All these dates were calibrated with OxCal using the IntCal13 calibration curve [33].

**Table 1:** ^14^C/AMS and Uranium series dates.

Sample	Number / location	Lab. code	Sample material	∂^13^C%o	pM	^14^C age BP	Calibrated yr BP IntCal13 (2σ)	Age group
CV-1	CV-1/Fig 5	CNA-3816.1.1	Carbonates	1	38,26±0.17	7,717±37	8,421–8,579	-
CV-2	CV-2/Fig 5	CAN-4165.1.1	Carbonates	1	2.25±0.07	30,480±258	33,979–34,899	-
CV–3	Z/A	CNA–669	Tooth *Hyaena*	-20,14		27,500±300	30,913–31,976	Adult
CV-5	End-scraper/Fig 6F	CAN-4176.1.1	Carbonates	-8.5	1.71±0.07	32,698±337	35,887–37,897	-
CV-14	CV-14/Fig 5	SU-182	Speleothem	-	-	15,318±851	(c. 17,020–13,616)*	-
CV-16	CV-16/Fig 5	SU-183	Speleothem	-	-	11,931±650	(c. 13,231–10,631)*	-
CV-17	CV-17/Fig 5	SU-184	Speleothem	-	-	11,543±633	(c. 12,809–10,277)*	-

Uranium and thorium content in calcite crusts and coatings specimens were determined for dating purposes [34]. Sample size was limited to a few milligrams (1-10mg/sample). Each sample, consisting of a thin calcite wafer, was cleaned and leached using pure water (18.2MΩ·cm-1), dissolved in nitric acid, spiked and digested in a closed vessel with HNO_3_, HCl and H_2_O_2_. After the sample digestion step, the solutions obtained were centrifuged in order to remove remains of silicates that could form part of the sample. Uranium and thorium separation and purification processes were performed by two step column resins (AG 1X8 and UTEVA), which allow higher precision measurements [35]. Chemical yields were better than 90%. Uranium and thorium concentrations were measured by isotope dilution analysis and isotope ratio determination was performed on a multicollector inductively coupled plasma mass spectrometer (MC-ICPMS, Thermo NEPTUNE). For increasing sensitivity, jet cones and membrane desolvation nebulizer (Aridus II) were used for all measurements. Neptune was operated at low resolution mode. Sample measurements were bracketed by dilutions of certified standard solutions. Spikes consisted of ^229^Th (SRM 4328c) and ^236^U (IRMM 3636).

Uranium concentrations of the measured samples varied (3–17 μg/g), while ^234^U/^238^U activity ratio was higher than 20 in two samples. Detrital contribution to U series results could be significant and ^230^Th/^232^Th activity ratios were lower than 10 for sample 14. Samples 16 and 17 present ^230^Th/^232^Th values around 20 so detrital correction should be small or absent. However, the calculation model led to inconsistent values for corrected ages; thus they were not reported. Results were calculated taking into consideration half lives stated [35, 36] and derived ages from results were finally obtained as shown in Table 2. Given the small sample amount and the detritus content, the derived ages should be taken with precaution.

**Table 2:** Uranium series analysis.

Sample ID	Lab. code	^238^U ng/g	^232^Th ng/g	^230^Th/^232^Th Atom x10-6	δ^234^U Measured	^230^Th / ^238^U Activity Ratio	^230^Th Age (yr BP) uncorrected
CV-14	SU-182	2 990 (21)	932 (19)	8 (0.1)	80.3 (5.0)	0.143 (0.014)	15,318±851
CV-16	SU-183	15 770 (106)	1 259 (25)	23 (0.1)	6.2 (0.5)	0.105 (0.011)	11,931±650
CV-17	SU-184	17 053 (32)	1 566 (32)	19 (0.1)	29.6 (1.5)	0.104 (0.010)	11,543±633

δ^234^U = 1000 [(^234^U/^238^U)—1]; δ^234^U_o_ = δ^234^U_meas_ · e^λ234·t^ BP: Before Present refers to before AD 1950. Uncertainties in parentheses by means of 2σ.

### Experimental approach

We also undertook an experiment by replicating the engraved motifs on fragments analogous to those of the parent rock surface on which the engravings were made. For this purpose, the analogous fragments were collected outside the cave. The experiment aimed to approach the technical requirements and the tools required for their execution in order to determine the method used to create the engravings and estimate the time invested in the task.

## Results

### Geomorphology

Ventanas Cave is located at 1,015 masl in an area which experienced a significant and fluctuating palaeoclimatic dynamic in the recent Quaternary period, documented in the nearby cave of Carigüela (Fig 1B call 2) [37, 38]. These climate conditions, together with the nature of the parent rock, created the surfaces that can be observed, where the engravings were done.

The interface between endo- and exokarst features is perfectly visible in Zone C, where the recession of the rock-shelter ceiling makes the sub-aerial exposure of this sector evident. Thus, the area that was most exposed to the exterior (away from the protection of the ceiling) began to develop an incipient karren, due to rainwater dripping from the overhang of the rockshelter ceiling.

It is important to highlight that the engravings must be older than the karren because the development of the karren has destroyed the engravings in several points. Besides, the sediments and vegetation removed for optimal observation of the engravings demonstrate that pedogenetic processes were taking place in Zone C. These observations undoubtedly indicate that the engravings are very old (thousands of years at least).

A second approximation of their age is provided by the weathering patinas that have been detected on some of the engravings in Zone C (Fig 5D–5F). The rainwater (dripping from the overhang of the rockshelter ceiling, for example) generated the formation of soluble bicarbonates (by the dissolution from atmospheric CO_2_ in rainwater falling on the limestone) which re-precipitated, in this case generating the weathering patinas. These patinas have been dated and characterized from a mineralogical point of view.

Finally, between the 16^th^ and 20^th^ centuries, anthropogenic activities in the areas outside Ventanas caused both the intense erosion of some areas and the loss of some fragments of the parent rock surface in Zone C. Of these actions, the greatest impact was caused by the construction of a wall in the area nearest the interior of the cave. This action largely preserved the engravings in Zone C, as these remained outside and therefore away from the influence of the activities carried out in areas nearer the interior (sheepfold, seasonal habitat, etc.).

### 3D modelling

A model of the surfaces with engravings was obtained with a maximum resolution of 5mm for distances below 50m in the case of the laser scanner, and 1mm for the handheld scanner. Therefore, geometric records of the areas, their orthogonal projections and further information can be included in a BIM model (Fig 4).

### Palaeolithic graphics

The identified graphic images are engravings located in the areas outside Ventanas (Zones A-B-C) (Figs 1C and 2) and, as such, they are exposed to natural light. However, the three entrances are oriented to the north, so direct sunlight only touches the panels with engravings just before sunset, when the sun, low in the sky, covers the engravings in light and shade. It is possible that these circumstances were a determining factor when choosing where to make the engravings.

**Fig 7:**
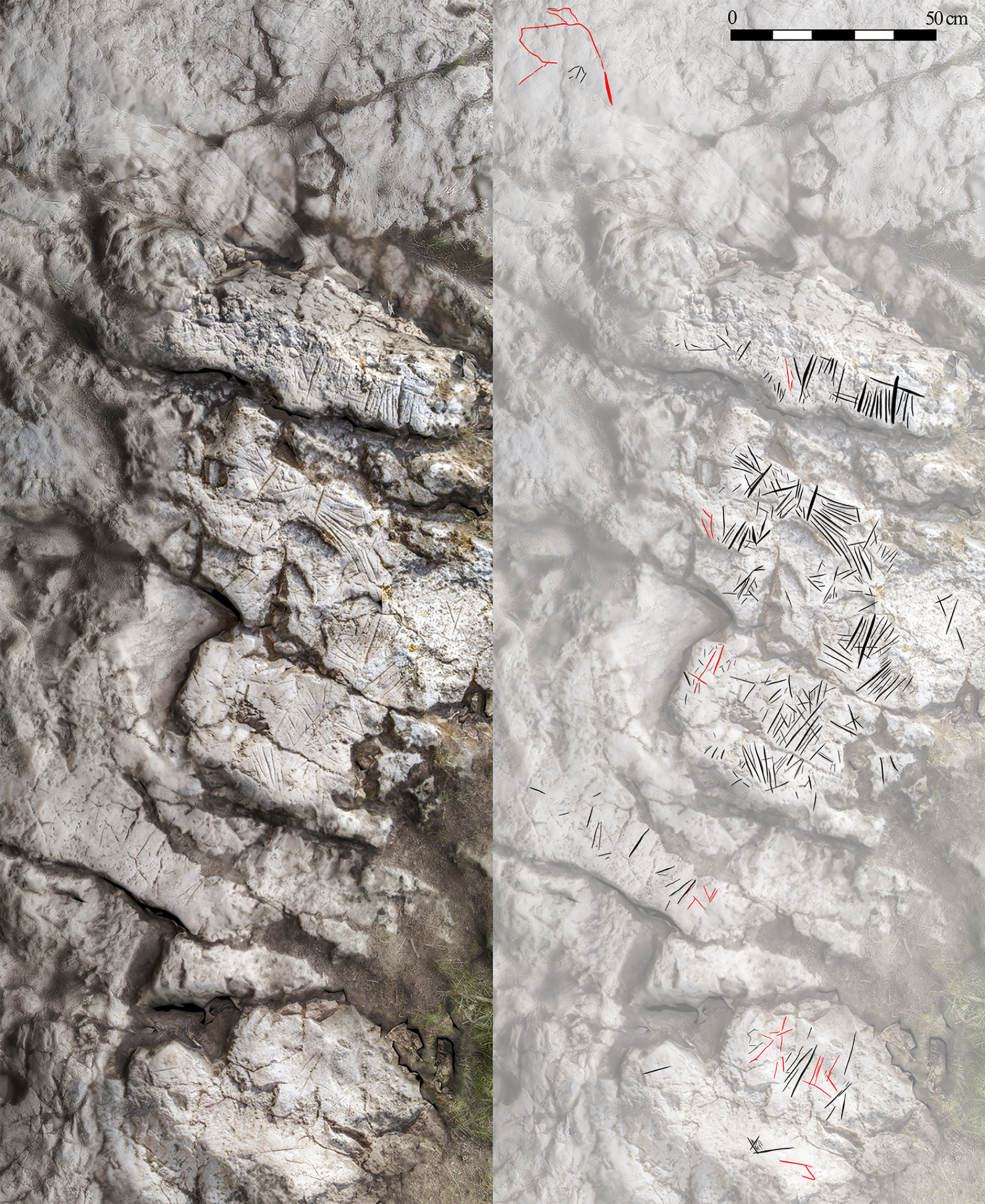
Zone C, main engravings area. Left, photorealistic render. Right, vectorized engravings with zoomorphs indicated in red.

In total, 765 V-shape section engraved lines with homogenous characteristics have been identified on the vertical wall plane and on the surface on the ground, in three different zones (Fig 7), as shown in Table 3.

**Table 3:** Engravings distribution by zones.

ZONE	A1	A2	B1	B2	C1	C2	C3	C4
>765 Engravings	-	22	71	50	5	>513	64	>40
% by subarea	-	2.9	9.3	6.5	0.7	67.0	8.4	5.2
% by Zone	2.9		15.8		81.3			

#### Zone A

**Fig 8:**
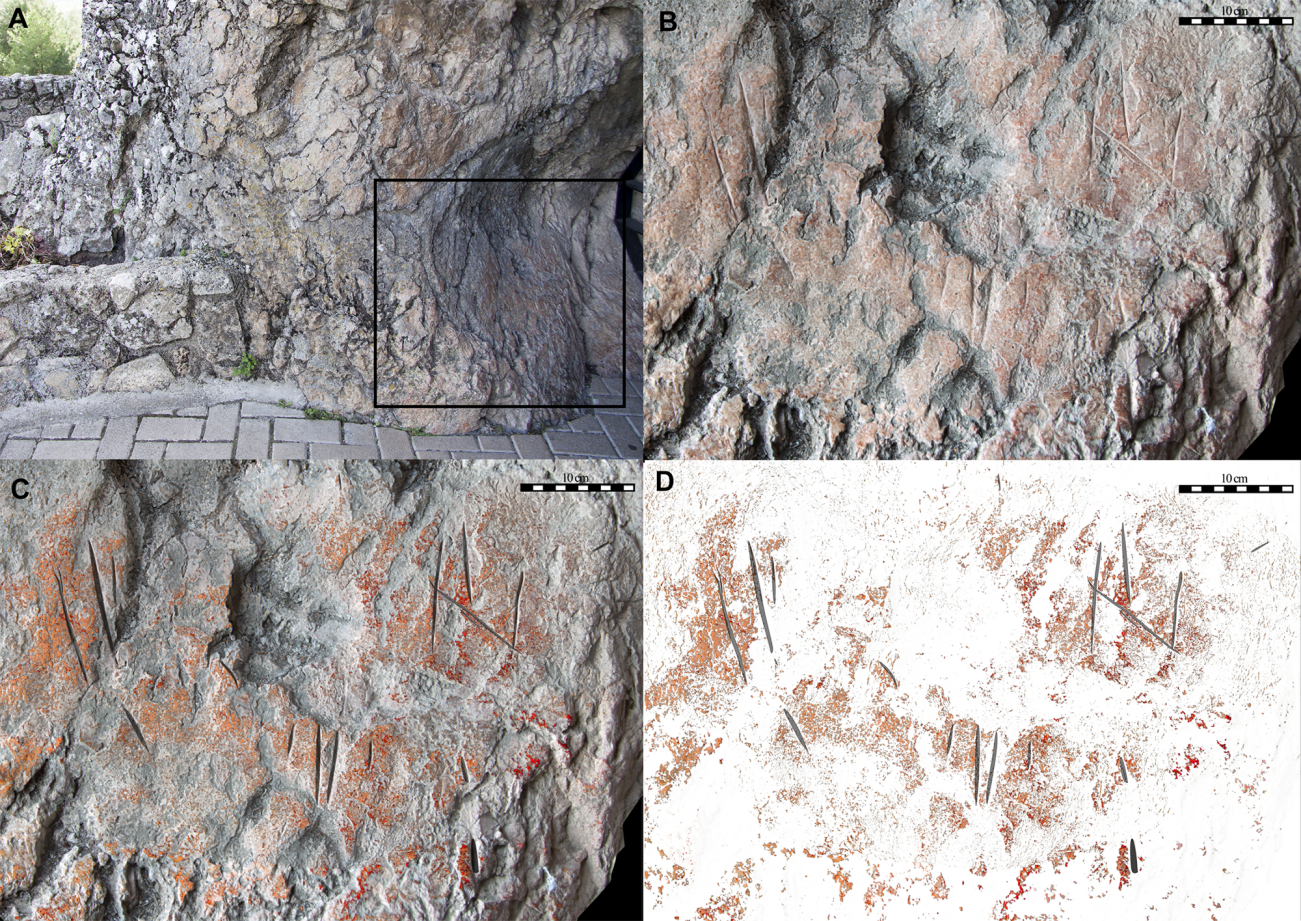
Zone A digital analysis. (A) Location. (B) Detailed photostitching. (C) Pigment classification using Principal Components Analysis. The two pigment concentrations levels identified are shown in orange (disperse) and red (dense). Vectorized engravings shown in black. (D) Pigments and engravings extraction.

The engravings are located in a 30x30cm area at the bottom left (+50cm from current ground level) of the vertical wall plane at the western entrance. The 22 engravings preserved (2.9% of the total engravings) mostly consist of deep, vertical lines (Fig 8).

#### Zone B

The engravings are to be found on both sides of the entrance, in the vertical wall plane. Those on the left are +50cm above current ground level, while those on the right are at +70cm. Another 121 engravings (15.8%) have been preserved here, which cover an area of 10x40cm.

#### Zone C

This 100x300cm area is the location of the largest concentration of engravings (622, 81.3%), carved into the ground. Currently, some of the engravings are not protected by the current ceiling, and are very near the escarpment that separates this area from the base of the slope, separated by an incline 3.5m in height. This is the result of the advancing erosion of the slope towards the interior of the karst. Therefore, Zone C is now most easily accessed via the interior of the cave, after entering through the central or western entrances. At the western edge a final group is found in a 20cm^2^ area (>40 engraved lines) and a few more at a short vertical distance from the ground. However, the considerable deterioration they have suffered means that, for the moment, we have not been able to obtain adequate documentation. This issue is also found in several ground-level areas, and above all a stalagmite with severely damaged patina remains (V-4).

The surfaces with the engravings were in some cases pre-treated by polishing the rock. Subsequently in some places red pigment, possibly diluted, was applied in the form of large patches, and finally the lines were engraved (Figs 7 and 8).

The results of the experiments suggest that the engravings were made with stone artefacts large enough to be gripped with the entire hand, thus enabling greater pressure to be applied to the surface-tool interface. To lubricate the motion of the tool and prolong the useful life of the edges, it is possible that this action was performed in a moist environment. The length of the linear, aniconic strokes seems to have possibly been conditioned by biomechanical factors; that is, the stroke length that can be made in a single stroke, without the artist releasing his/her hand, employing repetitive movements in the same direction in a to-and-fro motion. This goes some way to explaining both the average length of the strokes and the scarcity of corrections to the linear strokes.

**Fig 9:**
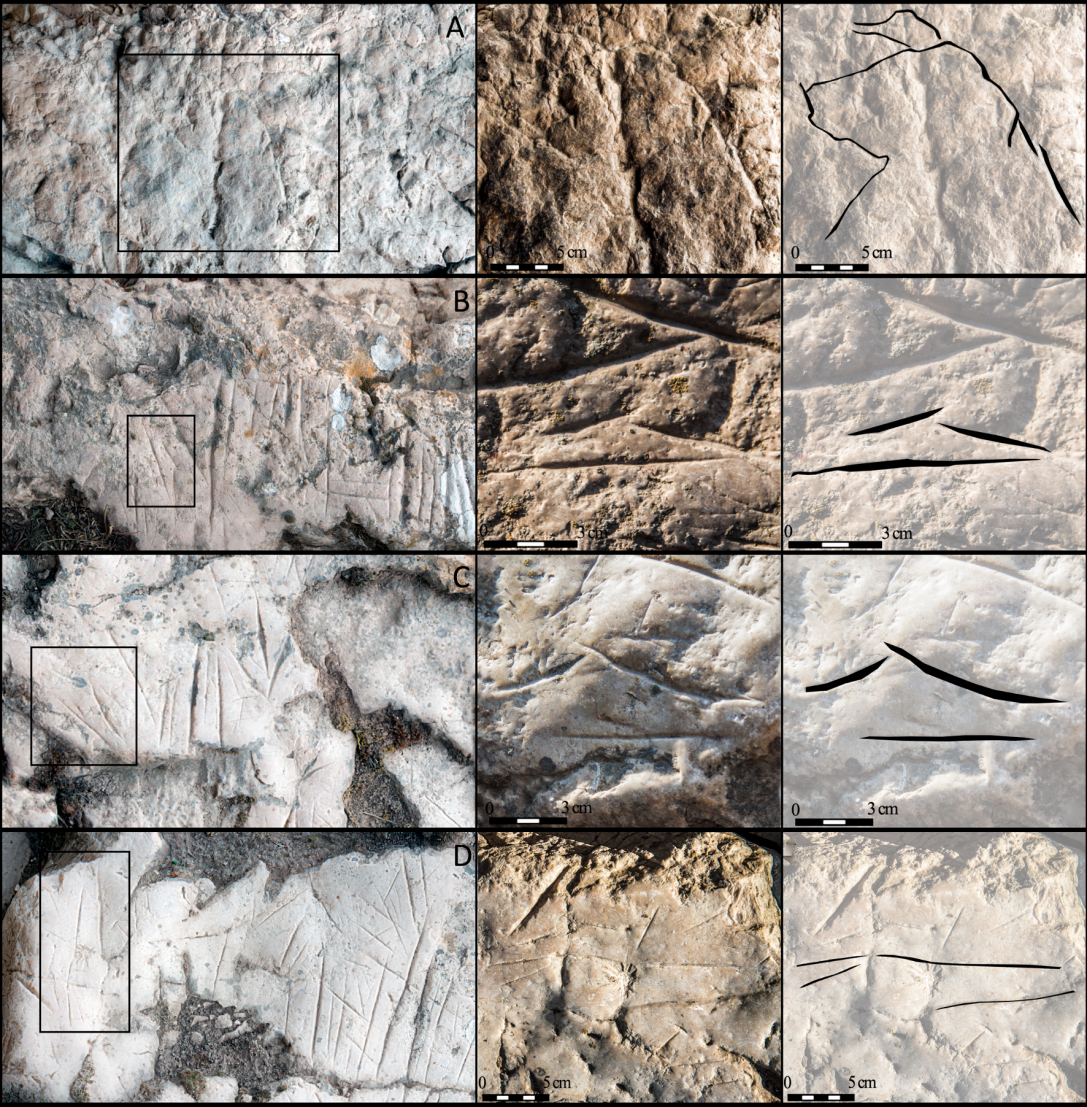
Details of the zoomorphs. (A) Auroch; B, C and D): Hinds.

**Fig 10:**
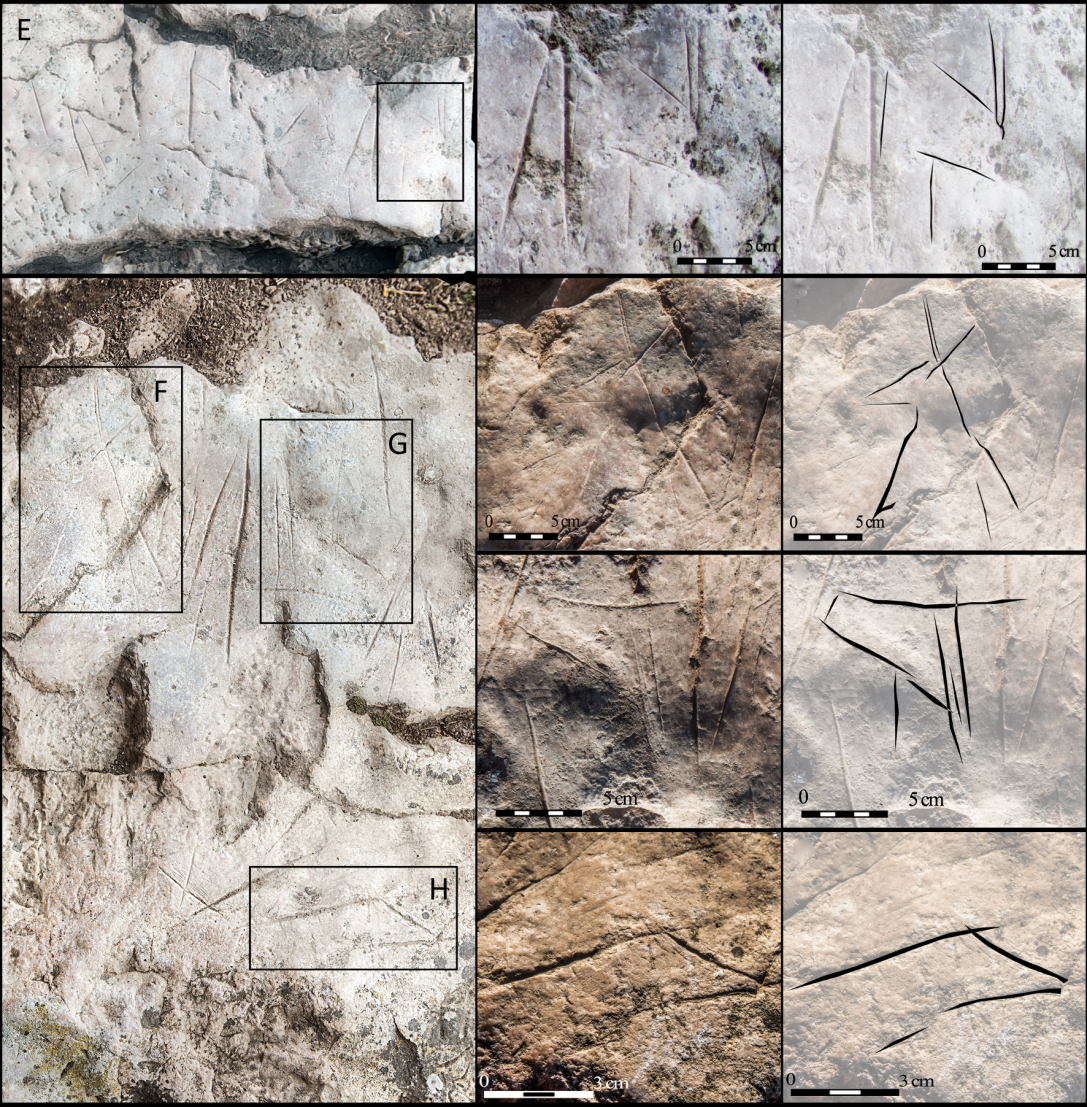
Details of the zoomorphs. E to H) Indeterminate animals.

The complete reading of the panels is made difficult by the loss/alteration of some areas of the support caused by landslides, limescale development and their physical and chemical deterioration. In other cases, there are parasite marks as a result of the execution of the various motifs, or scratches/erosion of the support created during the historic use of Ventanas. However, the preserved areas enable the engravings to be divided thematically into two groups or graphic horizons:

1. A first graphic ensemble, formed by non-figurative groups of linear or vertical strokes. Straight engravings of differing lengths up to 20cm. These are more than 1cm deep, although 10cm strokes are the most common, as they are not as deep as the former.
  At times, the lines appear to be grouped into series. In this group we include forms with a converging tendency ("angles") but which do not touch; in a few other cases they do form a corner, and in one case they slightly pass these angles. Another variant is "angles" divided internally by a line which never reaches the corner (“bisector”).
  At times, the intersection of lines produces aggregates that appear to be signs. However, in most cases, we discounted them due to the clear heterogeneity of the various lines that comprise the aggregates. Just in a few cases, they may indeed be signs, but these will be assessed in subsequent studies.
2. A second graphic horizon is located in Zone C, consisting of some incomplete zoomorphs, formed by engraving a reduced number of lines. To date, 8 zoomorphs have been identified (3 hinds, 3 aurochs, and 2 indeterminate) (Figs 9 and 10). They are all fore quarters between 23cm and 18cm in size, and generally occupy peripheral positions to the engraved lines of the first horizon.

### Characterization of pigments and weathering patinas

After Raman spectra baseline correction, calcite and dolomite were detected, corresponding to the natural support of the artworks, the bedrock. The presence of the most intense Raman bands for these compounds, calcite (1087, 713 and 282 cm^-1^) (http://www.ens-lyon.fr/LST/Raman/spectrum.php?nom=calcite) and dolomite (300 and 1096 cm^-1^) (http://www.ens-lyon.fr/LST/Raman/spectrum.php?nom=dolomite), along with quartz (206, 264, 354, 390, 450, 464, 697, 796, 808, 1069, 1162 cm^-1^[39], in all of the measured spots showed that the coloured layer is very thin.

Goethite (alpha-FeOOH) was the main mineral compound recorded by the Raman spectroscopy and was responsible for the red colour in the spots measured. The characteristic Raman spectrum of this mineral (goethite: 297, 384, 477 and 545cm^-1^) (RRUFF card Goethite_R050142 (780 nm laser) http://rruff.info/Goethite/R050142) is shown in Fig 5L compared with that of the recorded spectra in Panels 1, 2 and 4 (Fig 5G–5K). In all measurements the presence of hematite (bands at 226, 245, 292, 299, 411, 497 and 612cm^-1^) (RRUFF card Hematite_R040024 (785 nm laser) http://rruff.info/hematite/display=default/R040024) was also recorded as shown in Fig 5L.

The Raman spectra of the red coloured spots measured in this paper show a mixed signal from the bedrock and the pigment. The bedrock signal is dominated by calcite, dolomite and quartz; whilst goethite and hematite prevail in the pigment signal.

The mineralogical characterization of the weathering patinas observed over the engravings in Zone C shows two different groups (Table 4): on the one hand, V-1 to V-3 reflect calcite and dolomite in similar proportions, while V-4 and V-5 are dominated by the presence of calcite.

**Table 4:** Weathering patinas characterization.

Samples	Calcite (%)	Dolomite (%)	Quartz (%)
V-1	59	36	Traces
V-2	51	45	Traces
V-4	86	7	7
V-5	82	7	11

### Archaeology

In the sediments preserved in cracks and fissures in Zone C, 68 stone artefacts were recovered (Fig 6), of which 22 are retouched tools: 8 endscrapers (Fig 6A–6F, among others, 1 carinated and 4 nosed endscrapers), 4 burins (Fig 6G–6J, of which one is flat, (Fig 6I), 2 becs, 3 scrapers and 5 notches, as well as a hammer on a recycled blade core (Fig 6K). The techno-typological characteristics of this assemblage correspond to the EUP. Additionally, a hemimandible belonging to an *Ursus arctos* found in Zone C displays taphonomic marks indicating that it was processed anthropogenically (thermoalteration). Five hyena coprolites were also found. In another nearby area, Sector 2 (Fig 2), a stack of 70 hyena bone remains belonging to an MNI of 4 individuals, 3 adults and one infant, was documented [40].

### Chronology

According to ^14^C/AMS and U/Th dating of the weathering patinas (Tables 1 and 2), two groups of data can be observed. In the first group (CV-1, CV-14, CV-16 and CV-17) the samples cover from the Late Pleistocene (CV-14: 15,318±851 BP) to the Early Holocene (CV-1: 8421–8579 cal BP). The origin of this weathering patina could be related to the rainwater dripping from the rockshelter, and it probably began to be generated after the recession of the ceiling of the rockshelter.

In the second group (CV-2), the sample has yielded a much older age (CV-2: 33,979–34,899 cal BP). This sample was located outside the influence of the overhanging rockshelter, on the East wall, so the weathering patina has had a longer time to generate in this more open area.

As this weathering patina is covering some engraved lines, it is clear that in this zone they are older than 33,979–34,899 cal BP.

The third date was obtained by analysing the carbonates adhered to a carinated endscraper (Fig 6E). The resulting age (35,887–37,897 cal BP, Table 1) is within the development of the Evolved Aurignacian.

The technological, typometric and typological characteristics of the industries are very different from those that characterize the Solutrean or Magdalenian in southern Iberia [13]. The only exception is a possible lithic flake that might have originated from the making of a Solutrean tool, which was recovered from the sedimentary fill in the limestone pavement (Fig 6H) and, consequently, was deposited after the creation of the engraved lines affected by the karren. Finally, no artefacts clearly attributable to the Middle Palaeolithic have been identified in the lithic assemblages recovered in Ventanas [25].

The last hyena remains at Las Ventanas are located in Sector 2. In a carbonated context that seals the roof of the sequence, various bones belonging to this species were dated to ca. 30.9–32.0 cal ka BP (see Table 1). In this sense, the hyena coprolites recovered in Sector 1 can be correlated with the alternation of occupation between humans and this carnivore and the previous existence of exterior engravings, as would be demonstrated by the CV-2 carbonate date (33,979–34,899, 14C / AMS), before their burial.

### Experimentation

The observations made in the fieldwork and experiments enable us to identify a sequence consisting of: a) Polishing surfaces, for which it was necessary to use abrasive rocks with the aid of water; b) Colouring (at least partial) of surfaces with diluted red pigment; c) Engraving lines with both hands and using water to facilitate the process. The execution of each engraving requires several minutes of intense work. As a consequence, to make the >765 engravings preserved, which occupy an area of some 4m^2^, long sessions would have been necessary.

## Discussion

### Engravings

Technical, stylistic and typological parallels of many of the motifs can be found at sites on the Cantabrian coast, such as La Viña, Conde, Venta de La Perra, etc. [41, 42]. These manifestations are usually attributed historiographically to a chronocultural range within the Evolved Aurignacian and Gravettian, although for some authors they might date to the early phases of the Solutrean [1, 6, 9, 10, 20, 43].

The pre-Solutrean age of deep engravings, similar to those in Ventanas, has been demonstrated in Entrefoces rockshelter, where a block detached from the wall by cryofracturing was found stratified between Magdalenian levels. Stratigraphic, chronological and stylistic analysis showed that its execution was previous to the Lower Magdalenian and linked it to the external engraving horizon in the Nalón valley [44].

From a typological perspective, the two graphic horizons in Ventanas can be reduced to a few angular forms and the zoomorphs limited to the fore quarters of 3 hinds, 3 aurochs and another 2 indeterminate animals (Figs 9 and 10). The heads of some zoomorphs were depicted with the trilinear conventionalism observed at sites in the Cantabrian area. For example, this type of absolute profile view can be found in Chufín, Santo Adriano and La Viña.

### Period of execution and chronology

The >765 engravings preserved and identified in Ventanas do not seem to follow a single graphic plan. On the contrary, the final overall appearance seems to be more an accumulation of a series of motifs, probably designed over a long time period. Although we cannot discount the presence of a chronologically later motif, the available data suggest that most of the engravings can be attributed to pre-Solutrean times.

In order to approach the age of the engravings in Ventanas, it was necessary to resort to indirect information. Thus, the techno-typological traits of the lithic assemblage recovered in cracks and cavities in Zone C (Fig 6) seem to correspond to the EUP and, considering the typological value of the tools identified, they most closely match the Evolved Aurignacian. This ascription is also evidenced by the ^14^C/AMS age (35,887–37,897 cal BP) obtained from the carbonates adhered to a carinated endscraper (Fig 6E, Table 1).

As stated above, the technological, typometric and typological characteristics of the industries are very different from those that characterize the Solutrean or Magdalenian in southern Iberia [13] with the sole exception of a possible lithic flake that might have been produced when making a Solutrean tool. This was recovered from the sedimentary fill in the limestone pavement (Fig 6H) and, consequently, must have been deposited after the engravings had been affected by the karren. It should be stressed that no artefacts clearly attributable to the Middle Palaeolithic have been identified in the lithic assemblages at Ventanas [25].

Further indirect data to approximate the age of the lithic industries of Zone C is the presence of coprolites in association with them. In the context of the southern Iberian Peninsula, the last testimonies of this species come from contexts before the Late Glacial period [45, 46].

**Fig 11:**
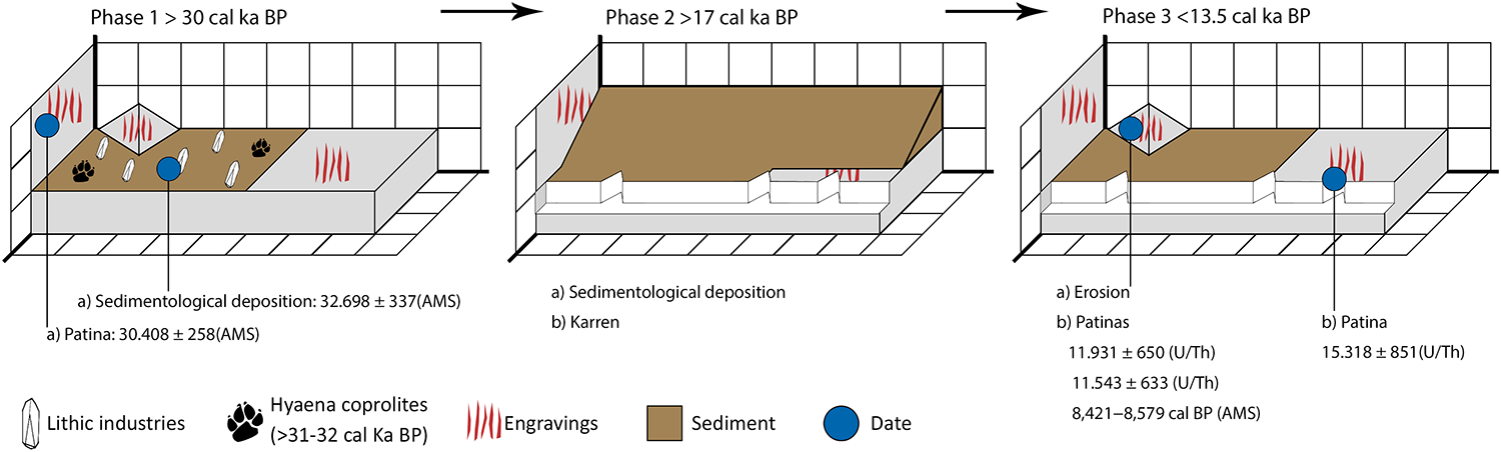
Visual synthesis of chronological and geomorphological sequence in Sector C at Cueva de Las Ventanas.

Subsequent (Fig 11) to the execution of some of the engravings, a patina was left that covered the edges of the limestone pavement and part of the pigments (Fig 5D–5F). It covered the areas with the analysed pigments (applied before the execution of the engravings) microns-thick, as shown by an analysis with Raman and short wave Infra Red spectra. In other cases, the patinas display a macroscopic growth, whose ^14^C/AMS dating gave an age of 33,979–34,899 cal BP (Table 1). Therefore, at least some of the first deep engravings in this area must have been made before that time.

Finally (Fig 11), it is worth noting that this reactivation of carbonate deposits on the karst during the range of ages obtained by the three ^14^C/AMS results (for the hyena jaw in Sector 2, patina 2 and carbonates on the carinated endscraper), which are within virtually the final stages of MIS 3, was also documented on numerous outcrops in southern Iberia and on the coast (Gibraltar), as well as in the inner mountain ranges (Sierra Morena and Sierra de Grazalema) [47, 48, 49, 50, 51].

The use of manganese and iron oxides by Neanderthals is well documented in Europe, especially for the period 60–40 ka BP [52, 53, 54]. In contrast, in Iberia, evidence of the use of mineral dyes is very limited in Middle Palaeolithic contexts [55, 56] and never on the scale seen in the Early Upper Palaeolithic. In this sense, although some recent work may [57] substantially change this perspective [58], we note that a) most of the Palaeolithic art in the SW of Europe is attributed to the Upper Palaeolithic (with abundant literature), b) the data obtained in Las Ventanas (archaeological remains and dates) lead us to propose that the ochre used on the surface, prior to the engravings, can be linked to the profusion of the use of pigments in Early Upper Palaeolithic levels in Europe or its application in graphic expressions [59] and c) there is no evidence supporting Middle Palaeolithic occupations at Las Ventanas. On the contrary, all the data we have obtained points to the Upper Palaeolithic. For this reason, we base ourselves on our data in order to avoid a speculative claim of a hypothetical Neanderthal graphic horizon.

Therefore, according these arguments, we can suggest that many of the engravings documented in Las Ventanas can be dated to the Evolved Aurignacian and Early Gravettian technocomplexes in the extreme southwest of Europe. This proposal has significant repercussions in the context of the debate on the MP-UP transition in southern Iberia, bearing in mind the similarity between Ventanas and the aforementioned sites with exterior engravings in the Cantabrian area, also correlated with the development of the EUP, and the proximity of Carigüela (Fig 1B), one of the key sites in the proposal of the late survival of the Middle Palaeolithic in southern Iberia [60]. Thus:

1. on the one hand, the graphic images at Ventanas support the idea of an early arrival of the EUP in southern Iberia, identified industrially and artistically for series attributed to the Evolved Aurignacian and Early Gravettian [1, 11, 18]; on the other hand, they serve to consolidate the idea that the innovations were not exclusively technological, but that they came together with all the associated complex symbolic expressions [1], including Palaeolithic art, in places like Ventanas, La Pileta and Ardales [16, 18, 19], among other sites [22].
2. these enable us to challenge chronologically the excessive survival of the MP in the south of Iberia, at least for the geographic context in which Ventanas is situated, above and beyond what has been proposed recently for the south of Iberia, *ca*. >43 ka cal BP [61].
3. as differences can be seen in the technical and stylistic traits of the graphic expression in Ventanas compared with other southern Iberian art ensembles, we suggest that, in the Evolved Aurignacian and Early Gravettian, there were several stylistic traditions in southern Iberia, and those which we see in Ventanas were either not widespread or there are more sites in the Mediterranean area that have still not been sufficiently assessed.
4. in sum, the absence of a Middle Palaeolithic record in Las Ventanas, the characteristics of the archaeological remains from the area adjacent to the engravings and the two dates obtained (for the carinated endscraper and one of the patinas: ca. 37.9–35.9 cal ka BP and 34.9–34.0 cal ka BP, Table 1) allow us to propose an age for the engravings at least contemporaneous with the development of the Evolved Aurignacian in the Mediterranean Iberian Peninsula. This technocomplex is currently well documented in various archaeological sites south of the Ebro river; for example and from south to north, Bajondillo (Málaga, Bj/11, ca. 39.5–32 cal ka BP) [13] Gorham’s Cave (Gibraltar, outer zone/context 9 with Upper Palaeolithic lithic and bone industries and ornaments, in ca. 34–33.2 cal ka BP [62], La Boja (Murcia, ca. 34–33.2 cal ka BP [63], Mallaetes (Valencia, level XII, 34.8–32.6 cal ka BP) and Cendres (Valencia, level XIVD, ca. 35.3–34.6 cal ka BP) [64].
5. additionally, the technical and thematic “proximity” between the hundreds of engravings at Ventanas and the isolated motif documented in Gorham’s Cave, attributed to the end of the Middle Palaeolithic [65], opens, with all due caution, a pathway to identifying possible interactions between modern humans and Neanderthals, a question that calls for future exploration.

## Conclusions

Ventanas (1,015 masl) is one of the highest altitude sites with Palaeolithic art in the whole of Iberia. The technique employed to produce the motifs was engraving, on the surface of the limestone in areas with sunlight, even though that sunlight only reached those areas directly at sunset.

The graphic images are largely concentrated at the westernmost entrance (Zone C/central area, with 67% of the motifs), while in the remaining areas the motifs are less numerous. Furthermore, they are limited to those areas nearest the vertical walls at both entrances (2.9% and 15.8%, Table 2).

The execution of the graphic images followed a process that involved: a) polishing the surface, b) colouring at least some areas with the application of ochre of local provenance but processed and applied possibly in the form of a solution, c) engraving the lines using medium/large stone tools.

The sequence of events that can be pieced together in Zone C is as follows: 1) execution of the engravings from at least the end of MIS 3 until the initial stages of MIS 2, articulated in two phases, an earlier one corresponding to the Evolved Aurignacian, and a more recent one attributed possibly to the Early Gravettian 2) weathering patina generation on the East wall (CV-2); 3) sediment deposit, preserved in cracks and crevices in which EUP-type tools are found; 3) development of an incipient karren probably due to the recession of the rockshelter ceiling; 4) weathering patina generation over the pavement in Zone C; 5) filling of the cracks with sediment and pedogenetic processes and 6) dissolution and loss processes of matrices linked to the use of the small cave during the Modern-Contemporary Age.

On a typological level, two graphic horizons appear: a first graphic horizon formed by non-figurative sets of linear or vertical strokes (grouped lines and "angles"), and a second graphic horizon with a few zoomorphs: isolated fore quarters of 3 hinds, 3 aurochs, and two indeterminate figures which occupy the periphery of the panels.

The entire ensemble of engravings at Ventanas displays similarities with the first and second graphic horizon in the Nalón valley in Cantabrian Palaeolithic art, attributed to a chronocultural range between the Aurignacian/Gravettian and Solutrean [10]. This similarity opens up intriguing expectations about the expansion of the EUP in Iberia or the existence of long-distance "relationships/contact" between the north and south of Iberia during this period.

In conclusion, the available chronological data and archaeological remains indicate that many of the engravings identified in Ventanas are from pre-Solutrean times. The most ancient stages begin, at least, with the Evolved Aurignacian, at ages of <35 cal ka BP, expressed mainly by linear deep engravings, while the trilinear conventionalism for drawing hinds is likely to have been established from Early Gravettian times and located around the previous ensembles.

Finally, the technical similarity of the linear engravings at Ventanas and an engraving attributed to the MP in Gorham’s Cave opens a door to the identification of relations between anatomically modern humans and Neanderthals before the latter finally disappeared definitively from southern Iberia.
