# Correction: Pre-Solutrean rock art in southernmost Europe: Evidence from Las Ventanas Cave (Andalusia, Spain)

**DOI:** 10.1371/journal.pone.0208212

**Published:** 2018-11-21

**Authors:** Miguel Cortés-Sánchez, José Antonio Riquelme-Cantal, María Dolores Simón-Vallejo, Rubén Parrilla Giráldez, Carlos P. Odriozola, Lydia Calle Román, José S. Carrión, Guadalupe Monge Gómez, Joaquín Rodríguez Vidal, Juan José Moyano Campos, Fernando Rico Delgado, Juan Enrique Nieto Julián, Daniel Antón García, M. Aránzazu Martínez-Aguirre, Fernando Jiménez Barredo, Francisco N. Cantero-Chinchilla

There are errors in the Funding section. The correct funding information is as follows: This study was sponsored by HAR2016-77789 (Spanish Ministry of Economy and Competitiveness) and 19434/PI/14 (Ministry of Education, Culture and Sport). For the fieldwork we had permission from the Andalusian Regional Government's Department of Culture. We also enjoyed the logistical support of the Píñar (Granada) Regional Government. This work was funded by the Andalusian Research Group Board RNM-349. "Proyecto de rehabilitación y conservación del yacimiento arqueológico "Cueva de las Ventanas" ("Restoration and conservation project for the 'Las Ventanas Cave' archaeological site"), Department of Culture, Andalusian Regional Government (File: BC.01.146/94.) "Análisis polínico y datación para Carbono 14 de huesos y coprolitos de hienas provenientes de la Cueva de las Ventanas depositados en el Museo Arqueológico y Etnológico de Granada ("Pollen analysis and Carbon 14 dating of hyena bones and coproliths originating in Las Ventanas Cave and desposited at the Archaeological and Ethnographic Museum of Granada"), Department of Culture, Andalusian Regional Government. File: BC 03/46/09). We received support from Ministerio de Economía y Competitividad, grant number CGL 2015–68604.
